# Determinants of morbidity and mortality related to health care-associated primary bloodstream infections in neonatal intensive care units: a prospective cohort study from the SEPREVEN trial

**DOI:** 10.3389/fped.2023.1170863

**Published:** 2023-05-31

**Authors:** Morgane Jaloustre, Robert Cohen, Valérie Biran, Fabrice Decobert, Richard Layese, Etienne Audureau, Nolwenn Le Saché, Marie Chevallier, Mohamed Riadh Boukhris, Pascal Bolot, Laurence Caeymaex, Manon Tauzin, with the SEPREVEN study Group

**Affiliations:** ^1^ Neonatal Intensive Care Unit, Centre Hospitalier Intercommunal de Creteil, Creteil, France; ^2^ Faculty of Medicine, University Paris Est Creteil, Creteil, France; ^3^ Groupe de Pathologie Infectieuse Pédiatrique, Paris, France; ^4^ Neonatal Intensive Care Unit, APHP, CHU Robert Debré, Paris, France; ^5^ Assistance Publique-Hôpitaux de Paris AP-HP, Hôpital Henri Mondor, Unité de Recherche Clinique (URC Mondor), Creteil, France; ^6^ University Paris Est Creteil, INSERM, IMRB, CEpiA Team, Creteil, France; ^7^ Pediatric Intensive Care and Neonatal Medicine, Bicêtre Hospital, AP-HP, Le Kremlin-Bicêtre, France; ^8^ Neonatal Intensive Care Unit, CHU Grenoble Alpes, Grenoble, France; ^9^ Department of Neonatology, CHU Lille, Lille, France; ^10^ Neonatal Intensive Care Unit, Centre Hospitalier de Saint-Denis, Saint-Denis, France

**Keywords:** bloodstream infections, newborn, outcomes, coagulase-negative staphylococci, preterm

## Abstract

**Background:**

Health care-associated primary bloodstream infections (BSIs), defined as not secondary to an infection at another body site, including central line-associated BSI, are a leading cause of morbidity and mortality in patients in neonatal intensive care units (NICUs). Our objective was to identify factors associated with severe morbidity and mortality after these infections in neonates in NICUs.

**Methods:**

This ancillary study of the SEPREVEN trial included neonates hospitalized ≥2 days in one of 12 French NICUs and with ≥ 1 BSI during the 20-month study period. BSIs (all primary and health care-associated) were diagnosed in infants with symptoms suggestive of infection and classified prospectively as *possible* (one coagulase-negative staphylococci (CoNS)-growing blood culture) or *proven* (two same CoNS, or ≥1 recognized pathogen-growing blood culture). BSI consequences were collected prospectively as *moderate morbidity* (antibiotic treatment alone) or *severe morbidity/mortality* (life-saving procedure, permanent damage, prolonged hospitalization, and/or death).

**Results:**

Of 557 BSIs identified in 494 patients, CoNS accounted for 378/557 (67.8%) and recognized bacterial or fungal pathogens for 179/557 (32.1%). Severe morbidity/mortality was reported in 148/557 (26.6%) BSIs. Independent factors associated with severe morbidity/mortality were corrected gestational age <28 weeks (CGA) at infection (*P *< .01), fetal growth restriction (FGR) (*P *= .04), and proven pathogen-related BSI vs. CoNS-related BSI (*P *< .01). There were no differences in severe morbidity and mortality between proven and possible CoNS BSIs. In possible BSI, *S. epidermidis* was associated with a lower risk of severe morbidity than other CoNS (*P *< .01), notably *S. capitis* and *S. haemolyticus*.

**Conclusions:**

In BSIs in the NICU, severe morbidity/mortality was associated with low CGA at infection, FGR, and proven pathogen-related BSIs. When only one blood culture was positive, severe morbidity/mortality were less frequent if it grew with *S. epidermidis* compared to other CoNS. Further studies to help distinguish real CoNS BSIs from contaminations are needed.

**Study registration:**

ClinicalTrials.gov (NCT02598609).

## Introduction

Neonates have a high risk of adverse events during their hospitalization in intensive care units (NICUs). Health care-associated infections (HAIs) are the most frequent adverse event in care (1). Extremely preterm neonates are particularly susceptible to HAIs, with a reported incidence around 40% (2, 3). Many studies have demonstrated the association between HAI risk and preterm birth or low birth weight (4–8).

Health care-associated primary bloodstream infections (BSIs), as defined by the US Centers for Disease Control and Prevention (9), are infections acquired at least 2 calendar days after admission and are BSIs that are not secondary to an infection at another body site. Because all BSIs in this study are primary and health-care associated, we will for simplicity's sake refer to them simply as BSI. Health care-associated primary BSIs account for more than half the proven neonatal HAIs (around 57%) (10) and are often associated with a central line (4, 11). Blood cultures remain the mainstay for the diagnosis of these BSI. Nonetheless, proven BSIs are often underestimated in neonates, especially in premature patients, due to blood sampling difficulties and their small intravascular blood volume. The blood volume sampled is often limited, and drawing blood for only one culture before starting antibiotic therapy is frequent.

In low birth weight neonates, sepsis is associated with a higher risk of mortality and morbidity, such as adverse neurodevelopmental outcomes and bronchopulmonary dysplasia, more ventilator days, and extended lengths of stay (6, 12–14).

The organisms involved in BSIs have been extensively described in developed countries: coagulase-negative *staphylococci* (CoNS) are responsible for 21% to 78% of HAIs, followed by Gram-negative bacilli (15%–33%), *Staphylococcus aureus* (8%–17%), *Enterococcus spp*. (3%–16%), and fungi (4%–12%) (6, 15–20). Nonetheless, the outcomes related to BSIs in NICU patients have not been fully described according to the organism involved and the patients’ characteristics. Studies have shown higher morbidity and mortality associated with Gram-negative compared to Gram-positive sepsis (6, 21, 22). Among preterm neonates, however, several studies have shown that CoNS BSI may also be associated with significant morbidity (12, 23, 24).

The SEPREVEN trial (25) was a stepped-wedge randomized controlled trial that evaluated the impact of an educational program on the rate of adverse events in NICUs. The main result of this trial was that a multiprofessional safety-promoting program in NICUs reduced the rate of adverse events. HAIs accounted for one of the most frequent categories of adverse events, and the trial collected outcomes related to these infections. The primary objective of this ancillary analysis was to identify factors associated with severe morbidity and mortality related to HAI BSI in neonates receiving intensive care. The secondary objectives were to describe the BSIs: the organisms involved and the risk factors associated with persistent HAI BSI.

## Materials and methods

### Study design and population

This was an ancillary study of the SEPREVEN trial (25), which took place between November 23, 2015, and November 2, 2017, in 12 French NICUs. Patients included in this study were hospitalized in one of the 12 participating NICUs for more than 2 days, with a corrected gestational age (GA) ≤ 42 weeks on admission, whose parents, after information, did not oppose use of their data, and who had one or more BSIs in the NICU during the SEPREVEN 20-month study period. We excluded cases where blood cultures grew two or more different commensal organisms or an unknown organism and cases where one blood culture grew a CoNS with a treatment that lasted less than 5 days.

### Data collection and definitions

Health-care associated primary BSIs were defined according to the Centers for Disease Control and Prevention 2015 definition as laboratory-confirmed BSIs that were not secondary to an infection at another body site (9). We included all health care associated primary BSIs in the study and, among them, we distinguished central line-associated BSI (CLABSI), according to the US Centers for Disease Control and Prevention 2015 definition: a laboratory-confirmed BSI with an eligible central line in place for more than two consecutive calendar days and on the date of event or the day before (9).

For descriptive purposes, we distinguished three types of BSIs: (i) BSIs with one blood culture growing a recognized bacterial or fungal pathogen (proven pathogen-related BSI); (ii) BSIs with clinical symptoms of infection and two or more blood cultures collected on separate occasions growing the same common commensal organism such as coagulase-negative *staphylococci* (proven commensal-related BSI); and iii) BSIs with only one blood culture growing a common commensal organism with clinical symptoms or signs compatible with infection and at least 5 days of antibiotic treatment (possible commensal-related BSI) (9). Because only one blood culture is often drawn before starting antibiotic treatment in neonates, we considered possible BSIs (6).

We included all health care associated primary BSIs in the study and we distinguished central line-associated BSI (CLABSI), according to the US Centers for Disease Control and Prevention 2015 definition: a laboratory-confirmed BSI with an eligible central line in place for more than two consecutive calendar days and on the date of event or the day before (9).

For each BSI, a physician, using a specific questionnaire, prospectively collected the following data: the patient's clinical characteristics and bacteriological data (clinical symptoms, organism involved, number and timing of blood samples, resistance status for Gram-negative *bacilli* and *S. aureu*s, time to positivity of blood culture). Patient's overall characteristics were collected prospectively within the SEPREVEN trial data collection.

Early removal of a central line was defined as removal on the day or day after antibiotic treatment started; all other cases were late removal or no removal. Persistent bacteremia was defined by a blood culture positive for the same organism after at least one day of antibiotic treatment.

### Outcomes

The physician prospectively graded the BSI consequences, according to the MCC MERP classification for medical error (26). Morbidity related to BSI was considered moderate if the consequence was only an antibiotic prescription (with or without use of noninvasive ventilation), or severe if the consequence included any of the following: a life-saving procedure (intubation, vasopressor drugs, chest compressions, or inhaled nitric oxide) and/or an extended length of stay in the hospital, and/or possible permanent damage (such as cystic periventricular leukomalacia), and/or contribution to death (26). Only the most severe consequence was collected.

### Analyses

Data are presented as medians [interquartile ranges, IQR] or means ± standard deviations (SD) for quantitative (continuous) variables and as numbers (percentages) for categorical variables. To compare characteristics between groups, we used Student *t*- or Mann-Whitney tests for quantitative variables and chi-square/Fisher exact tests for qualitative variables, as appropriate. To identify factors associated with severe morbidity/mortality and those associated with persistent bacteremia after initiation of antibiotic therapy, we conducted univariate and multivariate analyses using a mixed effect logistic regression model, with center and patient levels as random effects. The factors we studied were gestational age at birth, corrected gestational age at infection, sex, birth weight, type of BSI, FGR at birth (yes/no), time to positivity of blood culture, CLABSI, and catheter removal (early/late).

Factors associated with persistent bacteremia after initiation of antibiotic therapy were sought among the following: gestational age at birth, birth weight, sex, postnatal age at first positive blood culture (in days), time to positivity of blood culture (< or ≥ 12 h and < or ≥ 24 h), early removal of central line (yes/no), type of BSI, and presence of central line on the date of persistent positive blood culture (yes/no). Factors associated with the outcome at a *P*-value < .20 in the univariate analysis were entered into the multivariate analysis. We then applied a backwards stepwise approach to retain factors significant at the *P *< .05 level until we obtained a final model for each outcome.

The association between severe morbidity/mortality and the organism involved was studied for each type of BSI. All analyses were conducted with Stata software (v 16). A *P*-value of.05 was considered significant.

### Ethics

The SEPREVEN study was approved by the National Data Protection Authority (CNIL no 915263) and the relevant ethics committees (Consultative Committee on the Treatment of Data on Personal Health for research Purposes, France CCTIRS no 15327, and the Committee for the Protection of People Participating in Biomedical Research CPP Ile-de-France III, France, no ID RCB: 2014-A01751-46). The trial registration number was NCT 02598609. Parents were informed of data collection for the SEPREVEN study and did not oppose the use of their newborn's data.

## Results

### Population

**Figure 1:**
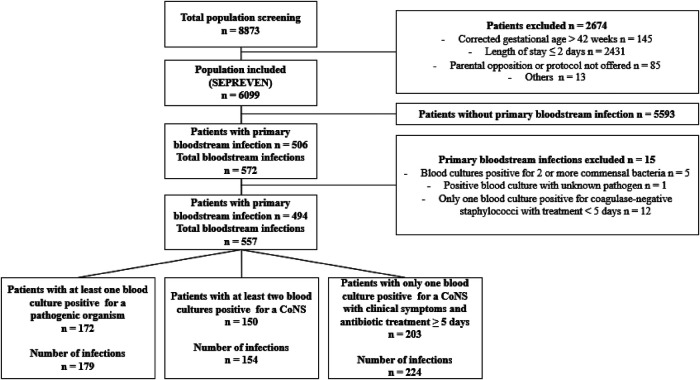
Flow chart. SEPREVEN, Study on preventing adverse events in neonates; CoNS, Coagulase negative *staphylococci*.

Among 6,099 patients included in the SEPREVEN cohort, 572 BSIs were collected in 506 patients. After 15 BSIs were excluded, the final study population comprised 557 BSIs in 494 patients (Figure 1). Table 1 presents the characteristics of the patients and the BSIs.

**Table 1:** Characteristics of patients and bloodstream infections.

Characteristics	*N* (%) or median (IQR)
N patients	494
Gestational age at birth, median [IQR]	27.6 [26–30.3]
<28 weeks	268/494 (54.2%)
28–31 weeks	154/494 (31.2%)
32–36 weeks	38/494 (7.7%)
≥37 weeks	34/494 (6.9%)
Age at time of infection (days), median [IQR]	10 [7–16]
Birth weight (g), median [IQR]	901 [730–1,250]
Sex (M/F)	267 (54.1%)/227 (45.9%)
FGR (<10th percentile)^a^	175/494 (35.4%)
Use of umbilical venous catheter during hospital stay	448/494 (90.7%)
N bloodstream infections	557
Number of blood cultures before antibiotic treatment (NA = 33)	
0	28/524 (5.3%)
1	385/524 (73.4%)
≥2	111/524 (21.2%)
Number of blood cultures positive after antibiotic treatment started (NA = 28)	222/529 (41.9%)
CLABSI	502/557 (90.1%)

IQR, interquartile range; FGR, fetal growth restriction; CLABSI, central-line associated bloodstream infection, NA, data not available.

^a^ According to Olsen's curves.

### Microbiological description

Common commensal organisms accounted for 378/557 BSIs (67.8%) and were mainly CoNS: 154/557 proven BSIs (27.6%) and 224/557 possible BSIs (40.2%). Recognized bacterial or fungal pathogens accounted for 179/557 (32.1%) BSIs (Table 2).

**Table 2:** Microbiological description of organisms involved in primary health care-associated bloodstream infections in the SEPREVEN cohort.

Total number of infections (%)	*N* = 557
**Proven pathogen-related BSIs**	**179** (**32.1)**
*Staphylococcus aureus* (MRSA *n* = 10)	66 (11.8)
Gram-negative bacilli^a^ (ESBL *n* = 2)	63 (11.3)
*Enterococcus faecalis*	19 (3.4)
*Bacillus (cereus n = 11/subtilis n = 1)*	12 (2.6)
*Candida (albicans n = 6/non-albicans n = 5)*	11 (2.0)
Group B *streptococcus*	4 (<0.1)
Other^b^	4 (<0.1)
**Proven commensal-related BSI**	**154** (**27.6)**
*Staphylococcus epidermidis*	83 (14.9)
*Staphylococcus capitis*	27 (4.8)
*Staphylococcus haemolyticus*	22 (3.9)
*Staphylococcus hominis*	9 (1.6)
*Staphylococcus warneri*	6 (1.1)
Other CoNS *(including 2 missing data)*	6 (1.1)
Other commensal organism^c^	1 (<0.1)
**Possible commensal-related BSI**	**224** (**40.2)**
*Staphylococcus epidermidis*	88 (15.8)
*Staphylococcus capitis*	54 (9.7)
Other CoNS	37 (6.6)
*Staphylococcus haemolyticus*	32 (5.7)
*Staphylococcus warneri*	7 (1.3)
*Staphylococcus hominis*	6 (1.1)

CoNS, coagulase-negative *staphylococci*; MRSA, Methicillin-resistant *Staphylococcus aureus*; ESBL, Extended-spectrum beta-lactamase.

^a^ Escherichia coli (*n* = 21), *Enterobacter cloacae* (*n* = 25), *Klebsiella pneumoniae* (*n* = 5), *Citrobacter other than freundii* (*n* = 1), *Enterobacter sakazakii* (*n* = 1), *Serratia marcescens* (*n* = 1), *Serratia liquefasciens* (*n* = 1), *Potoea dispersa* (*n* = 1), *Pseudomonas aeruginosa* (*n* = 4), *Pseudomonas non aeruginosa* (*n* = 1), *Acinetobacter baumanii* (*n* = 1), *Chryseomnas luteola* (*n* = 1).

^b^ *Corynebacterium afermentans* (*n* = 1), *Streptococcus bovis* (*n* = 1), classified as pathogenic organism by the clinician but organism not specified (*n* = 2).

^c^ Microbacterium sp.

Among possible commensal-related BSIs, ≥ 2 blood cultures were collected in only 32/224 (15.5%) cases.

### Morbidity and mortality after BSIs

Overall, moderate morbidity was found in 409/557 (73.4%) cases of BSI and severe morbidity/mortality in 148/557 (26.6%). The severe consequence was death in 35/557 (6.3%) BSIs, a life-saving treatment in 100/557 (17.9%) BSIs, an extended length of stay in 10/557 (1.8%), and possible permanent damage in 3/557 (0.5%).

### Factors associated with severe morbidity/mortality

Factors significantly associated with severe morbidity/mortality in the univariate analysis were a GA <28 weeks compared to 28–32 weeks (*P *= .02), a corrected GA <28 weeks at infection (*P *= .03), a birth weight <1,000 g (*P *= .02), and a recognized pathogen isolated in blood cultures (*P *= .03) (Table 3). In the multivariate analysis, factors independently associated with severe morbidity/mortality were a corrected GA <28 weeks at infection (*P *< .01), FGR (*P *= .04), and a proven pathogen-related BSI (vs. a proven or possible commensal-related BSI (*P *< .01) (Table 4). Concerning mortality alone in multivariate analysis, a corrected GA <28 weeks at infection (*P = *.04) and a proven pathogen-related BSI (*P = .*03) were significantly associated with mortality.

**Table 3:** Univariate analysis of factors associated with severe morbidity/mortality after health care-associated primary bloodstream infections.

Characteristics N (%) or (mean ± SD)	OR [95% CI]	*P*-value
GA at birth, weeks	0.95 [0.90–1.02]	.15
< 28	Ref	
28 to 31	0.51 [0.30– 0.89]	.**02**
32 to 36	0.55 [0.22–1.35]	.19
≥ 37	0.81 [0.33–2.04]	.66
Corrected GA at time of BSI (weeks)	0.93 [0.88–0.99]	.**03**
< 28	Ref	** **
28 to 31	0.57 [0.34–0.99]	**<**.**05**
32 to 36	0.33 [0.16–0.69]	**<**.**01**
≥ 37	0.60 [0.27–1.36]	.22
Sex		
Female	Ref	
Male	1.22 [0.78–1.91]	.39
Birth weight (g)		.**02**
< 1,000	Ref	** **
1,000 to 1,499	0.38 [0.20–0.71]	**<**.**01**
1,500 to 2,499	0.46 [0.19–1.11]	.09
≥ 2,500	0.91 [0.40–2.06]	.81
Type of BSI		.**03**
Proven pathogen-related BSI	Ref	
Proven commensal-related BSI	0.58 [0.31–1.07]	.08
Possible commensal-related BSI	0.44 [0.24–0.81]	**<**.**01**
CLABSI		
No	Ref	
Yes	1.01 [0.48–2.12]	.98
Time until blood culture was positive**^a^** (*n* = 285)		
< 12 h	Ref	
≥ 12 h	0.71 [0.38–1.32]	.28
< 24 h	Ref	
≥ 24 h	0.55 [0.24–1.28]	.17
Central line removal**^b^**		
Early removal	Ref	
Late or no removal	0.74 [0.38–1.46]	.39
FGR		
No	Ref	
Yes	1.45 [0.89–2.35]	.14

GA, gestational age; CLABSI, central-line associated bloodstream infection; FGR, fetal growth restriction; 95% CI, 95% confidence interval.

^a^ If more than one sample was taken for a blood culture before antibiotic treatment started, the shortest growth time was considered.

^b^ Analysis conducted on CLABSI (*n* = 502, 90.1%). Early removal: Day 0 or Day +1 of the start of the antibiotic therapy.

**Table 4:** Multivariate analysis of factors associated with severe morbidity/mortality of primary health care-associated bloodstream infections.

	Reference “Proven pathogen-related BSI“		Reference “Proven commensal-related BSI“		Reference “Possible commensal-related BSI“	
	OR [95% CI]	*P*	OR [95% CI]	*P*-value	OR [95% CI]	*P*-value
Corrected gestational age at time of BSI (weeks)		**<**.**01**	** **	** **	** **	** **
< 28	Reference					
28 to 31	0.53 [0.30–0.92]	.**03**	** **	** **	** **	** **
32 to 36	0.25 [0.11–0.56]	**<**.**01**	** **	** **	** **	** **
≥ 37	0.52 [0.23–1.18]	.12				
Type of BSI		.**01**		.**01**		.**01**
Proven pathogen-related BSI	Reference		1.77 [0.98–3.19]	.06	2.50 [1.37–4.57]	**<**.**01**
Proven commensal-related BSI	0.57 [0.31–1.02]	.06	Reference		1.41 [0.80–2.50]	.23
Possible commensal-related BSI	0.40 [0.22–0.73]	**<.01**	0.71 [0.40–1.25]	.23	Reference	
FGR	** **					
No	Reference					
Yes	1.68 [1.02–2.75]	.**04**				

BSI, bloodstream infection; FGR, fetal growth restriction; OR, odds ratio; 95% CI, 95% confidence interval.

The three multivariate models are the same, only the reference for the type of BSI is changed.

### Morbidity and mortality by type of organism

Among proven BSIs, risk of severe morbidity/mortality was similar among the different CoNS species (*P *= 0.23) and similar among the different bacterial or fungal pathogens (*P *= 0.64). A possible BSI with only one blood culture growing *S. epidermidis* was significantly associated with a lower risk of severe morbidity/mortality compared to the other CoNS (*P < *0.01) (Table 5).

**Table 5:** Clinical consequences of primary health care-associated bloodstream infections by organism.

	Severe morbidity/mortality n/N (%)	*P*-value*
Proven pathogen-related BSI		**.64**
*Staphylococcus aureus*	20/66 (30.3)	
Gram-negative bacilli	28/63 (44.4)	
*Enterococcus faecalis*	5/19 (26.3)	
*Bacillus*	3/12 (25)	
*Candida*	3/11 (27.3)	
Group B *streptococcus*	1/4 (25)	
Proven CoNS-related BSI	** **	**.23** **.60** ^a^
*Staphylococcus epidermidis*	19/83 (22.9)	
*Staphylococcus capitis*	4/25 (16)	
*Staphylococcus haemolyticus*	8/22 (36.4)	
*Staphylococcus hominis*	4/9 (44.4)	
*Staphylococcus warneri*	1/6 (16.7)	
Possible CoNS-related BSI	** **	**.06** **<.01** ^a^
*Staphylococcus epidermidis*	10/88 (11,4)	
*Staphylococcus capitis*	17/54 (31.5)	
*Staphylococcus haemolyticus*	9/32 (28.1)	
*Staphylococcus warneri*	2/7 (28.6)	
*Staphylococcus hominis*	2/6 (33.3)	

CoNS, coagulase negative staphylococcus; BSI, bloodstream infection.

Severe morbidity/mortality including extended length of stay or a probable permanent damage or a need for a life-saving treatment (intubation, inotropic treatment, chest compressions, or nitric oxide) or death.

* *P*-value adjusted for corrected gestational age at infection and FGR.

^a^ Analysis comparing staphylococcus epidermidis to other CoNS.

When considering only mortality, there was no significant difference among the different pathogens (*P *= 0.68) and among the different CoNS species in proven BSI (*P *= 0.97), respectively.

### Factors associated with persistent bacteremia

Among the 529 infections with available data about blood cultures after initiation of antibiotic treatment, bacteremia persisted in 222/529 (42.0%) cases. In multivariate analysis, after adjustment for corrected GA at infection and FGR, the only factor significantly associated with persistent bacteremia was the type of BSI: there was a higher risk of persistent bacteremia in proven CoNS-related BSI (OR, 7.22; 95% CI, 2.68–19.46], *P *< .01) and a lower risk of persistent bacteremia in possible CoNS-related BSI (OR, 0.22; 95% CI, 0.09–0.47], *P *< .01) compared to proven pathogen-related BSI. Early removal of the central line was not associated with a significant difference in the persistence of bacteremia (OR, 0.60; 95% CI, 0.22–1.61], *P* = 0.31).

## Discussion

This large prospective multicenter study including more than 500 BSIs in 12 NICUs reports both short-term and discharge outcomes. Because of the exhaustive and prospective multicenter data collection for the SEPREVEN trial, it offers a good overview of these particular infections for all GAs combined.

Among the 6,099 neonates included in the SEPREVEN trial, 8.1% presented a HAI primary BSI, with a risk inversely related to GA at birth. An original feature of our study was to distinguish primary BSI from other types of late-onset sepsis associated with specific locations. The incidence of all types of late-onset sepsis in our cohort was 18%, which is consistent with the literature (3, 27). Microbiological distribution in this cohort was also consistent with the literature, with a large predominance of CoNS, followed by *S. aureus*. and Gram-negative bacilli (6, 15–19). The largest share of CoNS species were *S. epidermidis* (45.2%), followed by *S. capitis* (21.8%) and *S. haemolyticus* (14.2%), again consistent with previous reports (20, 28).

The main result of our study was that severe infection-related morbidity/mortality was associated with a corrected GA <28 weeks at the time of infection, FGR at birth, and proven pathogen-related BSI. This study thus confirms the role of immaturity and growth restriction in neonatal outcomes in BSIs. It shows that risks factors of severity are not different in primary BSIs compared to late onset sepsis considered globally. This study also confirmed a higher risk of severe morbidity/mortality in BSI due to recognized pathogens compared to CoNS. Several studies in the literature have previously shown higher mortality in BSI with Gram-negative bacilli and fungi compared to Gram-positive bacteria (6, 21, 22). Our study also confirmed the considerable burden of CoNS BSIs, with severe morbidity found in 22.7% of cases, in line with the literature (29, 30). Our study found a low overall mortality rate (6.2%), little different from the rates found in the literature around 7% (7, 19), our large proportion of commensal-related BSI probably explained this result.

An original aspect of our study was its comparison of outcomes by CoNS species. We found that when only one blood culture was positive for *S. epidermidis*, neonates had a lower risk of severe morbidity than with other CoNS. This result raises two questions: is contamination responsible when only a single blood culture is taken and is positive? And does pathogenicity differ between CoNS species? To distinguish possible contaminants from true CoNS infections without underestimating the number of BSIs, we included CoNS infections with only one positive blood culture using criteria described in the literature (6) (one CoNS-growing blood culture with clinical symptoms and antibiotic treatment ≥ 5 days). In BSIs with only one positive blood culture, clinicians face a dilemma: whether to treat what might be a true BSI in a vulnerable patient or to protect her/him from possibly unnecessary antibiotic therapy (31). Taking samples for two blood cultures reduces the number of diagnoses of CoNS sepsis and thus the use of antibiotics (32).

Drawing culture samples from neonates, and especially very low birth weight neonates, can be technically difficult. In more than 2/3 of BSI cases, only one blood culture was available before antibiotic prescription: 385/557 (69%) regardless of the organism involved, and 168/224 (81.2%) in possible commensal-related BSI. If two blood samples had been collected for culture before antibiotic administration in all the cases, it would clearly have been easier to distinguish between real BSI and contamination.

The lack of a significant difference in morbidity/mortality in our study between neonates with a *proven* CoNS infection and those with *possible* CoNS infection suggests that many of the possible CoNS infections were true BSIs. Thus, other factors must be able to distinguish contaminants from true BSI. Time until the first culture is positive is likely to be the first clue in differentiating a contaminated sample from true BSI (33). When only one culture was positive for *S. epidermidis*, that time to positivity always exceeded 24 h. The second factor might be the particular CoNS species identified. The finding of similar rates of severe outcomes in proven and possible CoNS BSI for all CoNS species except *S. epidermidis* is interesting. *S. capitis* BSIs have been shown to be associated with higher morbidity in the literature than other CoNS infections (34). We might hypothesize that when only one blood culture is positive for a CoNS other than *S. epidermidis* (that is, for *S. capitis* or *haemolyticus*), it was more likely to be a true BSI than one positive for *S. epidermidis*.

Another interesting finding in our cohort was that removing the catheter at an early stage of infection did not appear to protect patients from severe morbidity/mortality. Removing the central catheter in cases of infection is controversial (35–37). An indication bias might exist in our cohort since one indication for removing the catheter could well be the patient's more severe symptoms. This might thus be a confounding factor with more severe infections in the early removal group. The Infectious Diseases Society of America (IDSA) recommends for both children and adults that the catheter be removed in cases of severe sepsis, suppurative thrombophlebitis, endocarditis, infections due to specific organisms (for instance, *S. aureus, P. aeruginosa,* or fungi), and when BSI continues despite 72 h of effective antimicrobial therapy (38). On the one hand, clinicians may feel reluctant to remove the catheter in neonates because of the difficulty in obtaining another venous access and the risk of bacterial dissemination at removal. On the other hand, possible severe consequences of BSI in this vulnerable population may motivate an early treatment and removal of the source of infection. Although our study might have lacked the power to reach a conclusion on this question, it does not support early removal of the catheter outside the severe situations described in adult guidelines.

In our study, cultures remained positive after starting antibiotic treatment more often for proven CoNS-related BSI than for proven pathogen-related BSI. This could be due to the ability of CoNS to form biofilms on medical devices such as central lines, thus providing a permanent source and continuous seeding of bacteria into the bloodstream (39, 40). Another explanation, however, might be the slow bactericidal activity of the vancomycin used to treat CoNS infections and the possible variability in vancomycin serum levels in patients. Finally, this finding could be due to the existence of contaminations in the possible commensal-related BSIs.

We had a higher proportion of persistent bacteremia in our cohort compared to other cohorts, which found persistence rates between 12% and 25% but it might be due to the large definition we took of persistent bacteremia (41–43).

Our study has several limitations. First, despite the high number of infections, we might have lacked power to distinguish differences in outcomes according to the organism responsible. Second, connecting clinical consequences, such as prolonged length of stay to a BSI episode can be difficult, as numerous factors may influence this length of stay. Clinicians were asked to report consequences prospectively after the infection, but assigning consequences to infections and not to prematurity or other conditions can be difficult and could have resulted in imprecision in our data. Nonetheless, these consequences were reported by senior physicians with experience in neonatal intensive care. Third, we lacked detailed data on type and doses of antibiotics used, and the appropriateness of antibiotic therapy could have influenced clinical outcomes. Fourth, blood sampling practices could have varied among participating NICUs, and we know that the volume of blood and the number of cultures are important for identifying BSIs. However, before starting the inclusions in the SEPREVEN trial, blood sample practices before antibiotic treatment were standardized in the participating units, by informing and encouraging health care professionals to collect two separate blood samples for culture, of a volume ≥ 1 ml. Finally, the educational program conducted during the SEPREVEN trial might have modified the incidence of CLABSI. However, it seems unlikely that this intervention changed the microbiological distribution or outcomes related to BSI that we report here.

## Conclusion

Independent factors associated with severe morbidity/mortality related to the health care-associated primary BSIs were a corrected GA <28 weeks at infection, a recognized pathogen causing the BSI, and FGR at birth. There were no differences in severe morbidity and mortality between proven and possible CoNS BSIs. When only one blood culture was positive, severe morbidity/mortality were less frequent if it grew with *S. epidermidis* compared to other CoNS. Further studies to help distinguish real CoNS BSIs from contaminations are needed, along with data on pathogenicity according to the organism involved and patients' characteristics.

## Data Availability

The original contributions presented in the study are included in the article/**Supplementary Material**, further inquiries can be directed to the corresponding author.
