# Supplementary material for: Determinants of morbidity and mortality related to health care-associated primary bloodstream infections in neonatal intensive care units: a prospective cohort study from the SEPREVEN trial
Source: Front Pediatr. 2023 May 31;11:1170863. doi: 10.3389/fped.2023.1170863 (PMC10264575; doi:10.3389/fped.2023.1170863)
Supplement: Supplementary file 1 [file Table1.docx]

**Supplemental data**

**Analyses**

Data are presented as medians [interquartile ranges, IQR] or means ± standard deviations (SD) for quantitative (continuous) variables and as numbers (percentages) for categorical variables. For comparisons of characteristics between groups, we used Student t or Mann-Whitney tests for quantitative variables and chi-square or Fisher's exact tests for qualitative variables as appropriate. To identify factors associated with severe morbidity/mortality, we conducted a univariate and a multivariate analysis using a mixed effect logistic regression model, with center and patient levels as random effects. The factors studied were gestational age at birth, corrected gestational age at infection, sex, birth weight, type of BSI, FGR at birth, time until the blood culture was positive, CLABSI, and catheter removal (early or late).

Factors associated with persistent bacteremia after initiation of antibiotic therapy were sought among the following: gestational age at birth, birth weight, sex, postnatal age at first positive blood culture (in days), time until the blood culture was positive (< or ≥ 12 hours and < or ≥ 24 hours), early removal of central line (yes/no), type of BSI, and presence of central line at the date of persistent positive blood culture (yes/no).

Factors associated with the outcome at the *P*<0.20 level in the univariate analysis were entered into the multivariate analysis. We applied a backwards stepwise approach to retain factors significant at the *P*<0.05 level until we obtained a final model for each outcome.

The association between severe morbidity/mortality and the organism involved was studied for each type of BSI. All analyses used Stata software (v 16), and a *P*-value <0.05 was considered significant.
